# The correlation between the triglyceride-glucose index and the odds of cervical cancer

**DOI:** 10.3389/fonc.2026.1766167

**Published:** 2026-03-12

**Authors:** Shanshan Li, Na Wang

**Affiliations:** 1 Department of Radiation Oncology, Zibo Municipal Hospital, Zibo, Shandong, China; 2 Department of Neurology, Zibo Municipal Hospital, Zibo, Shandong, China

**Keywords:** cervical cancer, insulin resistance, retrospective study, risk analysis, triglyceride-glucose index

## Abstract

**Background:**

Cervical cancer (CC) remains a major global health burden, particularly in developing regions. While HPV infection is the primary cause, metabolic factors like insulin resistance are increasingly implicated. The triglyceride-glucose (TyG) index, a marker of insulin resistance, has been linked to various cancers but its role in CC is underexplored.

**Methods:**

A preliminary exploration of the relationship between the TyG index and cervical cancer using the NHANES database, followed by validation of this association with data from Zibo Municipal Hospital and Shandong Cancer Hospital. TyG index was calculated and quartile-categorized. The association between the TyG index and cervical cancer was analyzed using logistic regression after adjusting for relevant covariates. Restricted cubic spline (RCS) curves were used to explore the non-linear relationship between the TyG index and cervical cancer odds, while ROC curves were employed to assess the diagnostic performance of the TyG index for cervical cancer.

**Results:**

Higher TyG index levels were significantly associated with increased odds of CC in both NHANES and hospital datasets. The association remained consistent across adjusted models. ROC analysis showed moderate diagnostic performance, especially for advanced-stage CC.

**Conclusion:**

Elevated TyG index were positively correlated with the odds of CC. Individuals with higher TyG index values should be vigilant about the increased odds of developing and progressing CC. Future studies are required to verify the association between them with larger-scale cohorts.

## Introduction

Cervical cancer (CC) remains a significant global health concern. According to the World Health Organization, cervical cancer is the fourth most common cancer among women, with approximately 604,000 new cases and 342,000 deaths reported in 2020 (1). The disease is primarily caused by persistent infection with high-odds types of human papillomavirus (HPV) (2). Despite screening and vaccination programs, the incidence of cervical cancer continues to rise, especially among women in developing regions, underscoring the need for improved odds identification and prevention strategies.

Current research indicates that several factors contribute to the etiology of CC, including HPV infection, sexual behavior, smoking and socioeconomic status (1, 3, 4). Recent studies have indicated that metabolic syndrome may play a crucial role in the pathogenesis of various types of cancer (5, 6). Metabolic syndrome is typically characterized by insulin resistance, which could contribute to a dysregulated hormonal environment conducive to tumor development.

The TyG index (Triglyceride-Glucose Index) is a marker calculated from fasting triglyceride (TG) and fasting blood glucose (FBG) levels, and is used to evaluate insulin resistance (IR).The triglyceride-glucose (TyG) index, a surrogate marker for insulin resistance, has been proposed as a potential biomarker for various metabolic disorders. In 2025, a Meta - analysis that included multiple prospective cohort studies found that an elevated TyG index was significantly associated with poor prognosis in patients with acute and chronic heart failure (7). The TyG index is independently associated with the progression of coronary artery calcification, especially in patients with diabetes, and its predictive ability is stronger than that of traditional risk factors (8). The TyG index and its derivative indices (such as TyG - BMI, TyG - WC) have shown high diagnostic accuracy in the screening and risk stratification of NAFLD, especially in primary healthcare settings with limited resources (9). In addition, the TyG index has also shown potential value in the field of tumor research. In gastric cancer, a high TyG index is associated with a higher tumor metastasis rate, chemotherapy resistance, and immunosuppressive state, suggesting that it can be used as a prognostic assessment tool (10). TyG - BMI (TyG×BMI) has a non - linear positive correlation with the overall survival (OS) of surgically resectable lung cancer patients (11). However, up to now, no study has explored the correlation between the TyG index and cervical cancer.

While the association between the TyG index and several types of cancer has been established (12), its significance in the context of CC remains insufficiently explored. Therefore, this study aims to explore the correlation between the TyG index and cervical cancer, filling the gap in this field.

## Methods

### Data source

We first selected the NHANES data from 2005-2016, and in our analysis, we included participants with comprehensive information on cervical cancer and TyG index. Initially, there were a total of 60936 participants. After excluding male participants (n=30152), participants with incomplete information and other types of cancer), our final analysis included 6092 eligible participants. Cervical-cancer cases were identified through the survey questions “Have you ever been told you have cancer?” and “What type of cancer was it?”. Among the participants, 6,005 had no history of cancer and 87 had previously been diagnosed with cervical cancer.

**Figure 1:**
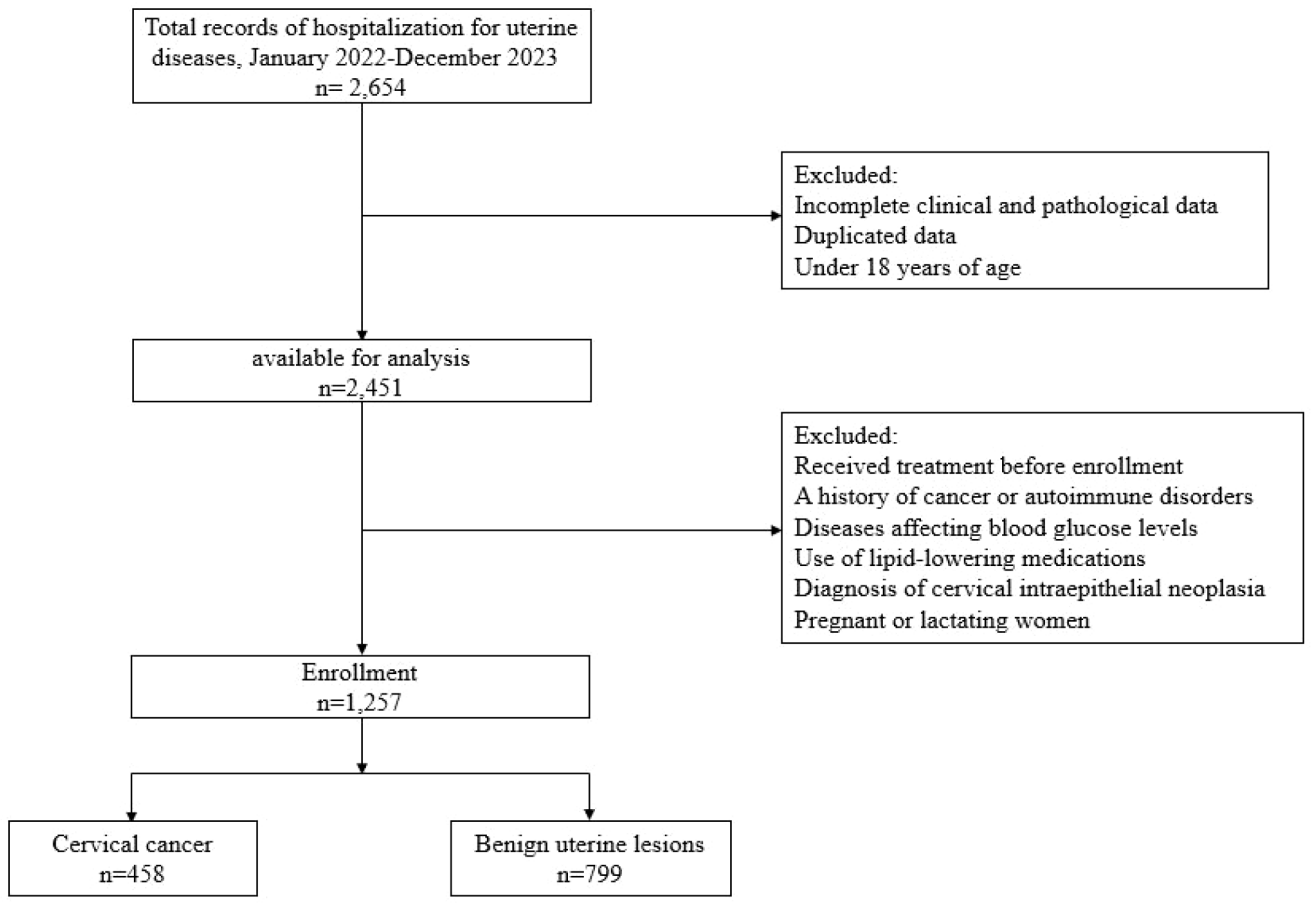
Flow chart of study subjects.

Our hospital data collection (**Figure 1**) was performed retrospectively from electronic medical records and hospitalization records at Shandong First Medical University Affiliated Shandong Cancer Hospital and Institute and Zibo Municipal Hospital including 2,654 patients with uterine diseases between January 2022 and December 2023. The study was approved by the Shandong First Medical University Affiliated Shandong Cancer Hospital and Institute’s ethics committee (approval number: SDTHEC2024010021) and Zibo Municipal Hospital’s ethics committee (approval number: 202401001), and informed consent was waived as anonymized sufferer records were used. The study cohort ultimately included 458 individuals with untreated CC diagnosed via pathological tissue biopsy and 799 subjects with benign uterine lesions, encompassing a total of 1257 participants enrolled in the research. Patients were excluded from the study based on the following criteria: (1) incomplete clinical and pathological data; (2) duplicated data; (3) under 18 years of age; (4) individuals who had undergone surgical treatment, chemotherapy, or radiotherapy prior to enrollment; (5) with a history of other malignancies or autoimmune diseases; (6) individuals with conditions that could potentially affect blood glucose levels, such as Cushing’s syndrome, hyperthyroidism, polycystic ovary syndrome, diabetes mellitus or pancreatitis; (7) use of lipid-lowering medications; (8) diagnosis of cervical intraepithelial neoplasia; (9) pregnant or lactating women.

### Covariables of NHANES

This study included a variety of covariates that may affect the relationship between the Tyg index and the oddss of cervial cancer. The variables considered include age, race, height, weight, education level, hypertension, alcohol consumption, total cholesterol (TC), triglycerides (TG), low-density lipoprotein cholesterol (LDL-c), Fasting plasma glucose (FPG), albumin and uric acid (UA).

### Information collection of hospital

Demographic data of participants were extracted from the hospital information system, encompassing age, height, weight, smoking status, alcohol consumption, marital status and medical history. The laboratory information system provided measurements for total cholesterol (TC), triglycerides (TG), high-density lipoprotein cholesterol (HDL-c), low-density lipoprotein cholesterol (LDL-c) and fasting blood glucose blood glucose (BG) levels.

### Definition of indicators

Body mass index (BMI) = weight (kg)/height squared(m2) (13). TyG index = Ln [TG (mg/dl) × BG (mg/dl)/2] (14).

### Statistical analysis

Data analysis was conducted utilizing SPSS, version 26.0. Parametric statistics, assuming normal distribution, were presented as mean ± SD, while non-parametric data were depicted using medians with interquartile ranges (M(P25, P75)). Group comparisons were performed either independent samples t-tests for normally distributed variables or Mann-Whitney U tests for those that deviated from normality. Categorical variables were tabulated as frequencies and percentages, with the chi-squared test employed to assess differences between patients with cervical cancer and controls. Logistic regression analysis was applied to identify potential predictors associated with cervical cancer. Restricted cubic spline (RCS) curves were used to explore the non-linear relationship between the TyG index and cervical cancer odds, while ROC curves were employed to assess the diagnostic performance of the TyG index for cervical cancer. All NHANES analyses were conducted using the appropriate survey weights. Statistical significance was set at the P<0.05 threshold.

## Results

### Baseline characteristics of the enrolled sufferers based NHANES

A total of 6,092 participants were included from the NHANES database, among whom 87 had cervical cancer and 6,005 were cancer-free. After weighted analysis, the two groups differed significantly across all included variables(p<0.001). The cervical-cancer cohort had markedly higher TyG index, triglycerides (TG), total cholesterol (TC), LDL-C, and body weight than the control group (**Table 1**).

**Table 1:** Baseline characteristics of the enrolled sufferers based on NHANES.

Variables	Total (n = 6092)	Cancer(-) (n = 6005)	Cancer(+) (n = 87)	*P*
Age, Mean ± SD	48.02 ± 17.49	48.06 ± 17.53	45.38 ± 14.17	<0.001
Glucose (mg/dl), Mean ± SD	105.03 ± 33.59	105.06 ± 33.66	102.70 ± 29.07	<0.001
Albumin (g/dl), Mean ± SD	4.12 ± 0.35	4.12 ± 0.35	4.13 ± 0.32	<0.001
UA (mg/dl), Mean ± SD	4.87 ± 1.30	4.88 ± 1.30	4.76 ± 1.01	<0.001
Tg (mg/dl), Mean ± SD	113.65 ± 63.24	113.45 ± 63.10	127.80 ± 71.40	<0.001
Tc (mg/dl), Mean ± SD	195.79 ± 41.20	195.78 ± 41.28	196.55 ± 35.81	<0.001
LDL-C (mg/dl), Mean ± SD	113.80 ± 35.47	113.77 ± 35.51	116.17 ± 32.77	<0.001
Height (cm), Mean ± SD	160.79 ± 7.13	160.77 ± 7.13	162.10 ± 6.47	<0.001
Weight (kg), Mean ± SD	76.41 ± 20.93	76.36 ± 20.92	80.21 ± 21.61	<0.001
Tyg index, Mean ± SD	8.52 ± 0.63	8.52 ± 0.63	8.61 ± 0.64	<0.001
Race, n(%)				<0.001
Mexican American	1012 (16.61)	1001 (16.67)	11 (12.64)	
Non-Hispanic Black	1286 (21.11)	1279 (21.30)	7 (8.05)	
Non-Hispanic White	2568 (42.15)	2508 (41.77)	60 (68.97)	
Other Hispanic	657 (10.78)	650 (10.82)	7 (8.05)	
Other Race - Including Multi-Racial	569 (9.34)	567 (9.44)	2 (2.30)	
Drinking, n(%)				<0.001
No	2425 (39.81)	2392 (39.83)	33 (37.93)	
Yes	3667 (60.19)	3613 (60.17)	54 (62.07)	
Hypertension, n(%)				<0.001
No	3963 (65.05)	3909 (65.10)	54 (62.07)	
Yes	2129 (34.95)	2096 (34.90)	33 (37.93)	
Educational level, n(%)				<0.001
High school and below	2804 (46.03)	2753 (45.85)	51 (58.62)	
Beyond high school	3288 (53.97)	3252 (54.15)	36 (41.38)	

CC, cervical cancer; TyG index, triglyceride-glucose index; BMI, body mass index; HDL-c, high-density lipoprotein cholesterol; TC, total cholesterol; LDL-c, low-density lipoprotein cholesterol; M, Median; Q, quartile; Z, Mann-Whitney test, χ², Chi-square test, -: Fisher exact.

### Baseline characteristics of the enrolled sufferers based on hospital

A total of 1257 sufferers were enrolled in the study, comprising 458 patients with CC and 799 controls (**Table 2**). There were no statistically significant differences in BMI, marital status and history of alcohol between the cervical cancer group and the control group (all P>0.05). The median TyG index was significantly different between the two groups, with values of 8.30 for the cervical cancer group and 8.64 for the control group (P<0.001). Additionally, there were statistically significant differences in age (P<0.001), TC (P<0.001), HDL-c (P<0.001), LDL-c (P<0.001), smoking history (P = 0.020) and history of hypertension (P<0.010) between the two groups.

**Table 2:** Baseline characteristics of the enrolled sufferers based on our hospital.

Variables	Total (n = 1257)	CC group (n = 799)	Non-CC group (n = 458)	*P*
Marital Status, n (%)				0.343
Yes	1246 (99.12)	790 (98.87)	456 (99.56)	
No	11 (0.88)	9 (1.13)	2 (0.44)	
Smoking, n (%)				0.020
Yes	7 (0.56)	1 (0.13)	6 (1.31)	
No	1250 (99.44)	798 (99.87)	452 (98.69)	
Dringking, n (%)				1.000
Yes	2 (0.16)	1 (0.13)	1 (0.22)	
No	1255 (99.84)	798 (99.87)	457 (99.78)	
Hypertension, n (%)				0.010
Yes	116 (9.23)	61 (7.63)	55 (12.01)	
No	1141 (90.77)	738 (92.37)	403 (87.99)	
TyG Index Groups (mg/dl), n (%)				<0.001
Q1 (<8.05)	306 (24.34)	254 (31.79)	52 (11.35)	
Q2 (8.05-8.42)	315 (25.06)	214 (26.78)	101 (22.05)	
Q3 (8.42-8.79)	318 (25.30)	189 (23.65)	129 (28.17)	
Q4 (≥8.79)	318 (25.30)	142 (17.77)	176 (38.43)	
Age/[M (P_25_, P_75_), years]	49.00 (43.00, 55.00)	47.00 (42.00, 52.00)	53.00 (45.00, 61.00)	<0.001
Height/[M (P_25_, P_75_), cm]	160.00 (157.00, 163.00)	160.00 (158.00, 164.00)	160.00 (155.00, 162.00)	<0.001
Weight/[M (P_25_, P_75_), kg]	60.00 (55.00, 67.50)	61.00 (56.00, 68.00)	60.00 (55.00, 66.00)	<0.001
BMI/[M (P_25_, P_75_), kg/m^2^]	23.80 (21.87, 26.08)	23.81 (22.03, 26.08)	23.76 (21.48, 25.97)	0.104
TyG Index, M (P_25_, P_75_)	8.42 (8.05, 8.79)	8.30 (7.97, 8.64)	8.64 (8.27, 8.98)	<0.001
TC/[M (P_25_, P_75_), mmol/L]	4.81 (4.20, 5.53)	4.75 (4.16, 5.37)	5.00 (4.26, 5.80)	<0.001
LDL-c/[M (P_25_, P_75_), mmol/L]	2.84 (2.37, 3.41)	2.74 (2.32, 3.24)	3.00 (2.50, 3.65)	<0.001

### The CC oddss and the TyG index based on NHANES

**Table 3** presents the relationship between the TyG index and cervical cancer based on NHANES data analysis. Model 1 was unadjusted. Model 2 was adjusted for age, race, educational level, height, weight and drinking. Model 3 was adjusted for hypertension, albumin, UA, TC and LDL-C based on Model 2. Across all three models, the Q4 group consistently exhibited the highest probability of cervical cancer. In Model 3, with the Q1 group as the reference, the odds of cervical cancer were 1.076-fold higher in the Q2 group (95% CI, 1.069–1.084; p < 0.001), 1.042-fold higher in the Q3 group (95% CI, 1.035–1.050; p < 0.001), and highest in the Q4 group, which showed 1.433-fold greater odds than Q1 (95% CI, 1.421–1.445; p < 0.001).

**Table 3:** Odds ratios for the association of TyG index with CC based on NHANES.

Variables	Model 1		Model 2		Model 3	
	OR (95%CI)	*P*	OR (95%CI)	*P*	OR (95%CI)	*P*
TyG Index Groups						
Q1 (<8.08)	1.00 (Reference)		1.00 (Reference)		1.00 (Reference)	
Q2 (8.08-8.50)	1.090 (1.083-1.097)	<0.001	1.040 (1.032-1.047)	<0.001	1.076(1.069-1.084)	<0.001
Q3 (8.51-8.94)	1.027 (1.020-1.034)	<0.001	0.980 (0.973-0.987)	<0.001	1.042(1.035-1.050)	<0.001
Q4 (≥8.95)	1.330 (1.321-1.339)	<0.001	1.223 (1.214-1.232)	<0.001	1.433(1.421-1.445)	<0.001

Model 1 was unadjusted. Model 2 was adjusted for age, race, educational level, height, weight and drinking. Model 3 was adjusted for hypertension, albumin, UA, TC and LDL-C based on Model 2.

CC, cervical cancer; TC, total cholesterol; LDL-c, low-density lipoprotein cholesterol; TyG index, triglyceride-glucose index; OR, Odds Ratio, CI, Confidence Interval.

### The CC odds and the TyG index based on hospital

**Figure 2:**
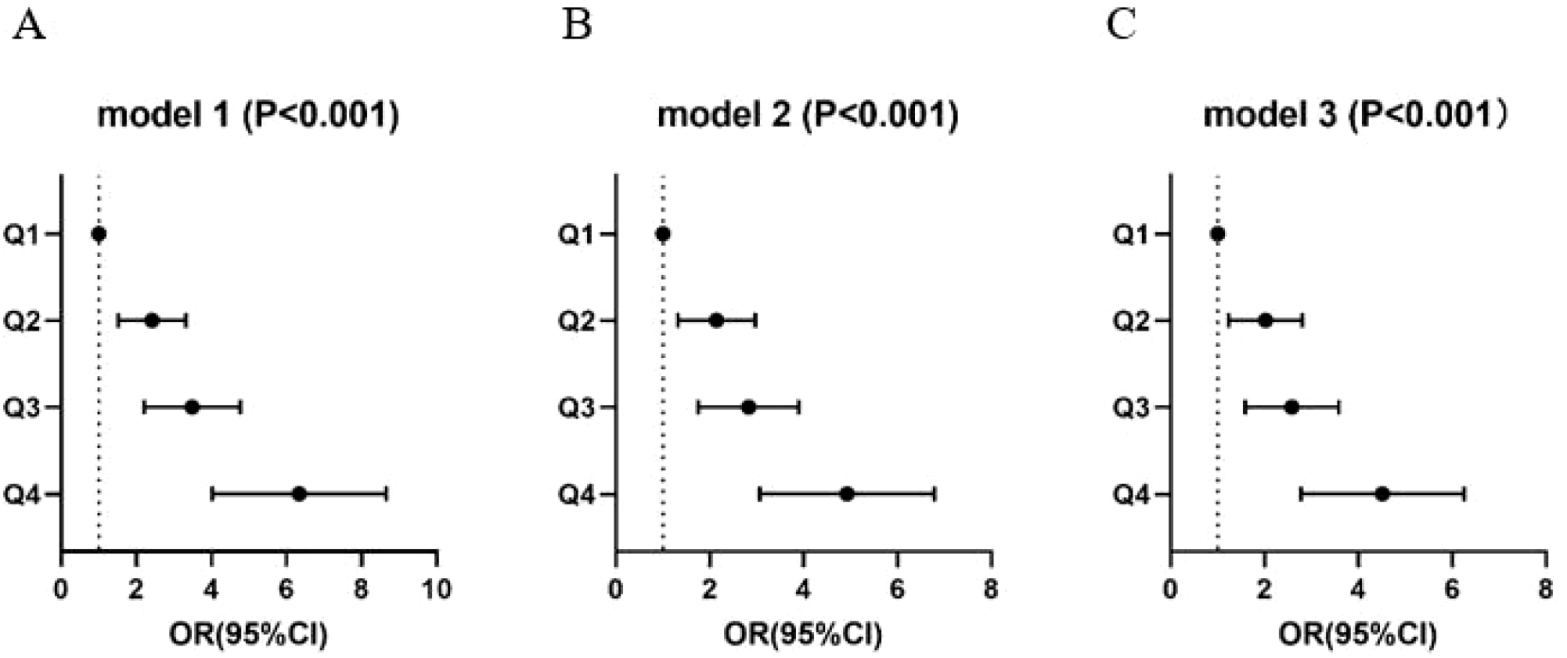
The relationship between the TyG index quartiles and CC. **(A)** unadjusted; **(B)** adjusted for age; **(C)** adjusted for age, smoking, hypertension, TC and LDL-c. CC, cervical cancer; TyG index, triglyceride-glucose index; TC, total cholesterol; LDL-c, low-density lipoprotein cholesterol; OR, odds ratio.

**Table 4** and **Figure 2** illustrate the association between CC odds and the TyG index. The odds correlation between TyG index quartiles and CC odds is delineated in **Table 2**. In the unadjusted Model 1, individuals in Q2, Q3 and Q4 quartiles of the TyG index exhibited a higher CC odds compared to the Q1, with corresponding oddss ratios (OR) and 95% confidence intervals (CI) of 2.31(1.58-3.37), 3.33(2.30-4.84) and 6.05(4.18-8.78), all with P<0.001. After adjustment for age in Model 2, the Q2, Q3 and Q4 of the TyG index also showed an increased adjusted odds compared to Q1, with adjusted OR (95% CI) of 1.99(1.35-2.94), 2.63(1.79-3.85) and 4.61(3.15-6.76), all with P<0.001. Similarly, in Model 3, the TyG index was identified as a odds factor for CC, with odds increasing as TyG levels increased. Compared to the Q1 group, the odds was 1.92 times higher in the TyG Q2 group (95%CI, 1.29-2.85, P = 0.001). The TyGQ3 group had a 2.46 times higher odds compared to Q1 (95%CI:1.66-3.64, P<0.001). Individuals in the Q4 group had an even higher odds, being 4.29 times that of the Q1 group (95%CI:1.29-2.85, P<0.001). Model 3 included additional confounding factors such as age, smoking, hypertension, TC and LDL-c.

**Table 4:** Odds ratios for the association of TyG index with CC based on our hospital.

Variables	Model 1		Model 2		Model 3	
	OR (95%CI)	*P*	OR (95%CI)	*P*	OR (95%CI)	*P*
TyG Index Groups						
Q1 (<8.05)	1.00 (Reference)		1.00 (Reference)		1.00 (Reference)	
Q2 (8.05-8.42)	2.31 (1.58-3.37)	<0.001	1.99 (1.35-2.94)	<0.001	1.92 (1.29-2.85)	<0.001
Q3 (8.42–8.79)	3.33 (2.30-4.84)	<0.001	2.63 (1.79-3.85)	<0.001	2.46 (1.66-3.64)	<0.001
Q4 (≥8.79)	6.05 (4.18-8.78)	<0.001	4.61 (3.15-6.76)	<0.001	4.29 (2.90-6.35)	<0.001

Model 1 was unadjusted. Model 2 was adjusted for age. Model 3 was adjusted for age, smoking, hypertension, Total cholesterol and LDL-c.

CC, cervical cancer; TC, total cholesterol; LDL-c, low-density lipoprotein cholesterol; TyG index, triglyceride-glucose index; OR, Odds Ratio, CI, Confidence Interval.

### Diagnostic characteristic of the TyG index for CC

**Figure 3:**
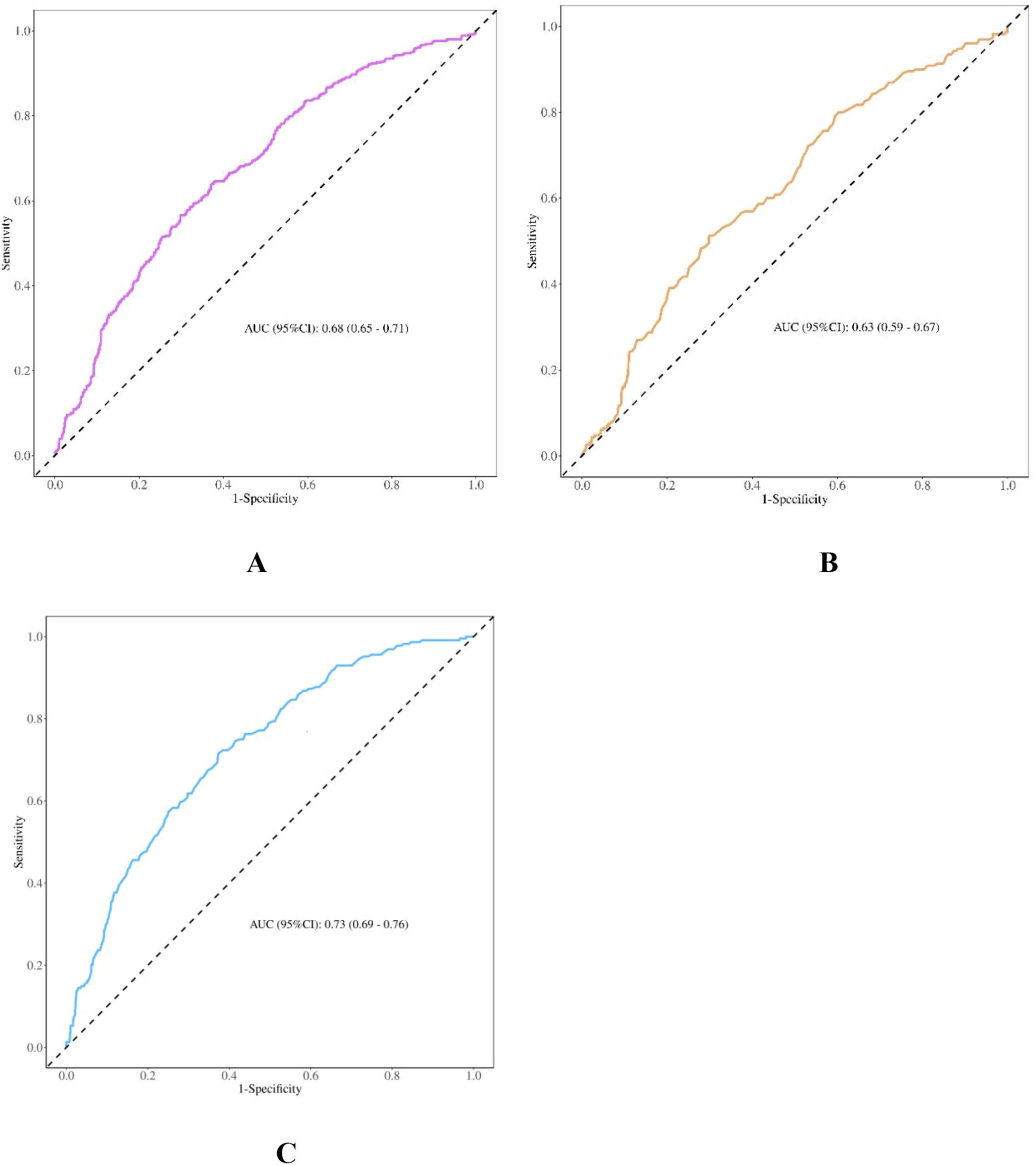
ROC of the TyG index in differentiating between benign and malignant cervical diseases. **(A)** ROC analysis of the TyG index in patients with benign cervical lesions versus all-stage cervical cancer patients. **(B)** ROC analysis of the TyG index in patients with benign cervical lesions versus stage I+II cervical cancer patients. C: ROC analysis of the TyG index in patients with benign cervical lesions versus stage III+IV cervical cancer patients.

**Figure 3** and **Table 5** present the ROC curves of the TyG index in distinguishing between benign and malignant cervical diseases. The optimal threshold for the TyG index in detecting CC was identified as 8.57, with an area under the curve (AUC) of 0.68 (sensitivity 0.70, specificity 0.57) (**Table 3**, **Figure 3A**). The stages of CC are dichotomized into early (stages I+II) and advanced (stages III+IV) stages. For early-stage cancer, the AUC was 0.63(sensitivity 0.70, specificity 0.51) (**Table 3**, **Figure 3B**). Notably, the TyG index for advanced cancer exhibited a larger AUC than the other groups, at 0.73(sensitivity 0.63, specificity 0.70) (**Table 3**, **Figure 3C**), all P<0.001.

**Table 5:** Diagnostic characteristic of the TyG index for CC.

A			B			C		
AUC (95%CI)	Sensitivity (95%CI)	Specificity (95%CI)	AUC (95%CI)	Sensitivity (95%CI)	Specificity (95%CI)	AUC (95%CI)	Sensitivity (95%CI)	Sensitivity
								(95%CI)
0.68 (0.65-0.71)	0.70 (0.67-0.73)	0.57 (0.52-0.61)	0.63 (0.59-0.67)	0.70 (0.67-0.73)	0.51 (0.45-0.58)	0.73 (0.69-0.76)	0.63 (0.59-0.66)	0.71 (0.6-0.77)

(A) ROC of the TyG index in differentiating between benign and total population of cervical cancer; (B) ROC of the TyG index in differentiating between benign and early stages of cervical cancer; (C) ROC of the TyG index in differentiating between benign and advanced stages of cervical cancer.

CC, cervical cancer; ROC, receiver operative characteristic; TyG, triglyceride-glucose; AUC, area under the curve; CI, confidence interval.

### Sensitivity analysis

Using data from our hospital, we did a sensitivity analysis after excluding all patients who have hypertension, smoke, or drink alcohol. The results show that for every unit increase in the TyG index, the odds ratio (OR) of developing cervical cancer is 3.009 (95%CI, 2.291-3.951, P<0.001). This indicates that our results are stable.

### Non-linear relationship between the TyG index and the odds of cervical cancer

**Figure 4:**
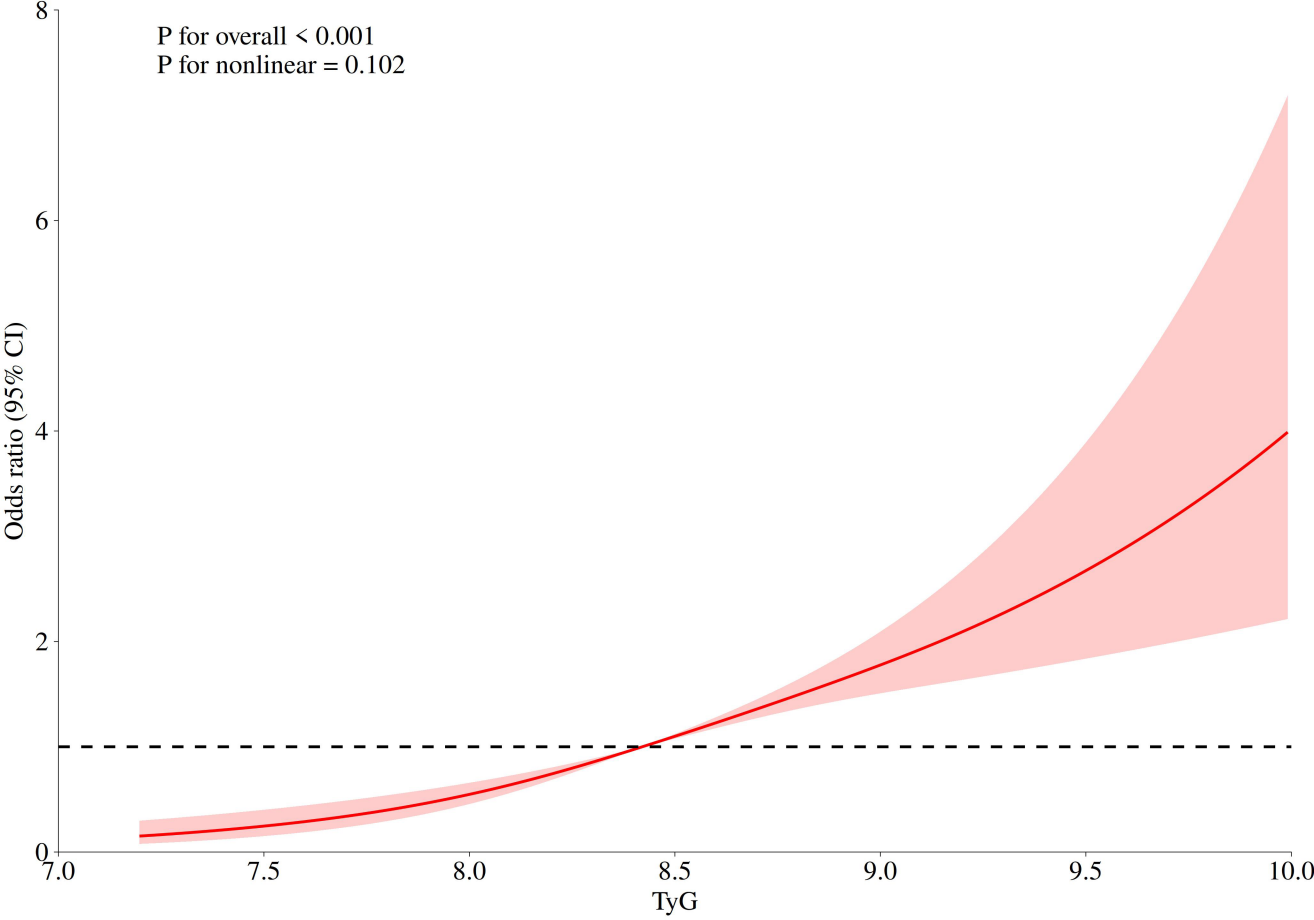
RCS curve.

We used RCS curves to examine the nonlinear relationship between the TyG index and the odds of cervical cancer. The results showed (**Figure 4**) that the TyG index exhibited a linear relationship with the odds of cervical cancer (P for nonlinear = 0.102).

## Discussion

CC remains a significant global health challenge, ranking as the fourth most common malignancy among women worldwide (15). The primary etiological factor associated with CC is persistent infection with high-odds HPV types, particularly HPV 16 and 18, leading to the development of precancerous lesions and invasive carcinoma (16). The disease often has a prolonged asymptomatic phase, with symptoms typically arising in advanced stages, including abnormal vaginal bleeding, pelvic pain, and systemic manifestations in cases of metastasis. Additionally, several modifiable odds factors such as smoking, multiple sexual partners and immunosuppression contribute to the disease’s incidence and progression, necessitating ongoing research into prevention and early detection strategies (17). In light of the increasing incidence of cervical cancer and the limitations of current screening and diagnostic methods, our study investigates the potential role of the TyG index as a non-invasive biomarker for assessing cervical cancer odds. Previous literature has established the correlation between insulin resistance, as indicated by the TyG index and various malignancies, including breast cancer, colorectal cancer, gastrointestinal cancer and so on (18–20). For example, Several studies have shown that the TyG index is significantly associated with the risk of breast cancer, and that people with a TyG of 8.9 or higher are 2.17 times more likely to develop breast cancer (21–23). In addition, the TyG index is significantly linked to both the development of gastric cancer and the prognosis for patients with it (24–27). Moreover, recent studies have also demonstrated that the TyG index is significantly associated with the onset and progression of liver and colorectal cancers (28, 29).

This is the first study focusing on TyG index and cervical cancer in Asian women. Our findings demonstrate that the TyG index is significantly elevated in cervical cancer patients compared to those with benign cervical diseases, suggesting its potential utility in early detection and odds stratification. The TyG index, which stands for triglyceride-glucose index, is significant for assessing metabolic health. Compared with current insulin resistance and inflammation indicators (HOMA-IR, hs-CRP, SCC-Ag, NLR/PLR, etc.), the TyG index can be calculated with just a simple blood sugar and fat test, without the need for complicated calculations. In addition, TyG index does not rely on insulin measurement, does not require multiple inflammatory markers, and is not interfered by obesity indicators. Nevertheless, it maintains similar diagnostic accuracy, enabling low-cost risk assessment in resource-limited settings. In places with limited resources, it can replace multiple biomarker tests, providing a ‘low-cost, high-information’ way to screen for risks (30–32).

The underlying mechanisms through which the TyG index may exert its carcinogenic influence in the progression of CC are multifaceted and not yet fully elucidated. The TyG index is an alternative marker for insulin resistance (IR). IR is characterized by a diminished responsiveness of cells and tissues to the peptide hormone insulin. Hyperinsulinemia, a hallmark of IR, leads to reduced hepatic production of insulin-like growth factor binding proteins 1 and 2 (IGFBP-1/2), thereby increasing the tissue bioavailability of free IGF-1 and IGF-2. This enhancement in turn augments the activation of intracellular mitogenic signaling pathways and disrupts fundamental metabolic pathways in precancerous and malignant cells (33–36). Furthermore, hyperinsulinemia may also impact the hepatic production and secretion of sex hormone-binding globulin, increasing the bioavailability of sex steroids (estradiol and testosterone), which can positively influence tumorigenesis and tumor progression (37, 38). In addition, inflammatory cells in adipose tissue of individuals with insulin resistance may promote systemic inflammation, creating an environment conducive to tumor growth. Excessive reactive oxygen species in insulin-resistant patients may also damage DNA, promoting mutations and carcinogenesis (37).

The rising incidence of CC underscores the importance of identifying modifiable odds factors and high-odds populations to mitigate its impact on public health. Our study introduces the TyG index as a potential non-invasive biomarker for assessing CC odds. The significant difference in TyG index levels between CC patients and those with benign cervical diseases suggests that the TyG index could play a crucial role in early screening and intervention strategies. This aligns with findings from previous studies that highlight the importance of metabolic parameters in CC assessment, indicating that elevated TyG index could be indicative of underlying insulin resistance, which is a known contributor to cancer progression (39, 40).

Moreover, our comprehensive analysis reveals a positive correlation between increasing TyG index quartiles and the odds of CC, reinforcing the notion that metabolic dysfunction is intricately linked to cancer pathogenesis. Specifically, our data demonstrate that individuals in the highest TyG quartile exhibit a significantly elevated odds of developing CC, even after adjusting for confounding factors such as age, smoking, TC and LDL-c. Our results suggest that the TYG index has potential to predict the likelihood of developing cervical cancer, which has not been extensively addressed in the literature (12). However, we need bigger studies in the future. They should keep exploring and validating our findings.

## Conclusion

In conclusion, this study confirms that there is a significant association between the triglyceride-glucose index (TyG index) and the odds of cervical cancer, suggesting that this index also has the potential to identify populations at high risk of cervical cancer. However, due to its lack of specificity relative to other cancers, it cannot be used as a specific biomarker. In addition, the results of this study have not yet confirmed a causal relationship between the two. Future studies should be conducted in more diverse populations and different regions with larger sample sizes to further explore the association between the TyG index and the risk of cervical cancer.

### Limitation

The limitations of this study must be acknowledged to provide a comprehensive understanding of the findings. Firstly, the design constrains the ability to establish causal relationships between the TyG index and CC odds, limiting the interpretation of temporality. Additionally, the reliance on self-reported demographic and lifestyle factors may introduce recall bias and affect the accuracy of the data collected. Furthermore, our sample size, while adequate for initial associations, may not capture the full spectrum of variations in TyG index across different demographics. The number of cervical cancer (CC) cases in the NHANES cohort is quite small (n=87), which might limit how effective subgroup analyses can be. Future studies should aim to increase the number of cervical cancer cases, especially early-stage cervical cancer cases, to make subgroup analyses more reliable. The diagnostic accuracy of the TyG index is only moderate, and further efforts are needed to explore and improve its diagnostic performance in the future. As our study population was entirely hospital-based, our findings may not be generalizable to the broader community.

Future research should focus on longitudinal studies that can better elucidate the temporal relationship between TyG index fluctuations and cervical cancer development, as well as more multicenter prospective trials that include diverse populations to enhance the robustness of our conclusions.

## Data Availability

The raw data supporting the conclusions of this article will be made available by the authors, without undue reservation.
